# Bioinspired Stabilization of Amorphous Calcium Carbonate by Carboxylated Nanocellulose Enables Mechanically Robust, Healable, and Sensing Biocomposites

**DOI:** 10.1021/acsnano.2c12385

**Published:** 2023-03-22

**Authors:** Wanlin Wu, Zhixing Lu, Canhui Lu, Xunwen Sun, Bing Ni, Helmut Cölfen, Rui Xiong

**Affiliations:** 1 State Key Laboratory of Polymer Materials Engineering, Polymer Research Institute of Sichuan University, Chengdu 610065, China; 2 Engineering Research Center of Polymer Green Recycling of Ministry of Education, College of Environmental and Resource Sciences, Fujian Normal University, Fuzhou 350007, China; 3 Physical Chemistry, Department of Chemistry, University of Konstanz, Konstanz 78457, Germany

**Keywords:** amorphous calcium carbonate, biomineralization, nanocellulose, biocomposites, mechanical properties

## Abstract

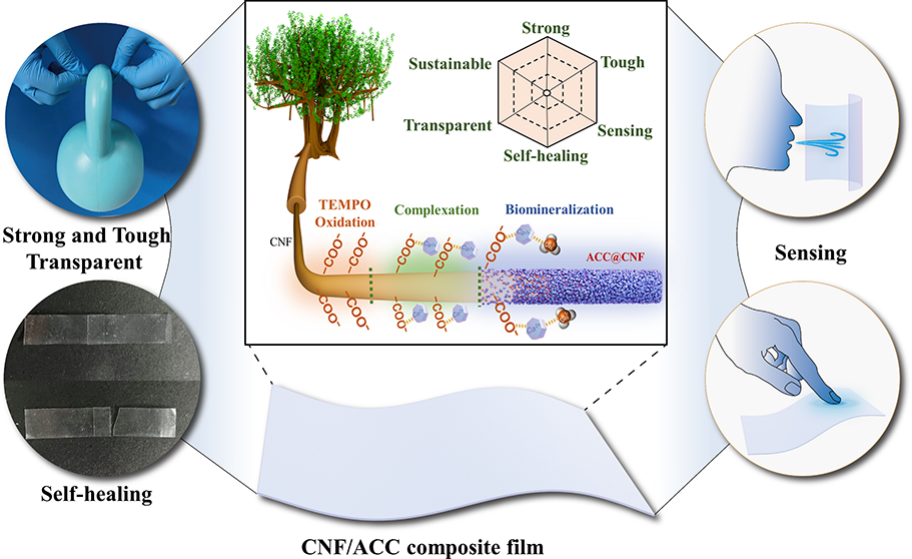

Nature builds numerous structurally complex composites with fascinating mechanical robustness and functionalities by harnessing biopolymers and amorphous calcium carbonate (ACC). The key to successfully mimicking these natural designs is efficiently stabilizing ACC, but developing highly efficient, biodegradable, biocompatible, and sustainable stabilizing agents remains a grand challenge since anhydrous ACC is inherently unstable toward crystallization in the wet state. Inspired by the stabilized ACC in crustacean cuticles, we report the efficient stabilization ability of the most abundant biopolymer–cellulose nanofibrils (CNFs) for ACC. Through the cooperative stabilizing effect of surface carboxyl groups and a rigid segregated network, the CNFs exhibit long-term stability (more than one month) and achieved a stabilization efficiency of 3.6 and 4.4 times that of carboxymethyl cellulose (CMC) and alginate, respectively, even higher than poly(acrylic acid). The resulting CNF/ACC dispersions can be constructed into transparent composite films with the high strength of 286 MPa and toughness up to 28.5 MJ/m^3^, which surpass those of the so far reported synthetic biopolymer-calcium carbonate/phosphate composites. The dynamic interfacial interaction between nanocomponents also provides the composite films with good self-healing properties. Owing to their good wet stability, the composite films present high humidity sensitivity for monitoring respiration and finger contact.

## Introduction

Living creatures are capable of building damage-torrent structural materials with on-demand functionalities from a well-organized biopolymeric matrix and biominerals.^1^ These biominerals are usually crystalline with specific shapes arranged into ordered arrays as observed in teeth and nacre.^2,3^ However, the anisotropic mechanical properties of crystalline minerals usually cause vulnerable mechanics of natural structural materials in certain directions. Additionally, molding crystalline minerals into desired micro/nanostructures to add functionalities also faces great difficulties due to the large size, anisotropic shapes, and surface chemistry of crystalline minerals. Living creatures therefore evolve an alternative strategy of utilizing amorphous minerals as intermediate/permanent building blocks to achieve structurally complex materials at the micro/nano length-scale as well as isotropic mechanics.^4^ For example, sponge spicules are made of amorphous silica or amorphous calcium carbonate (ACC) with well-defined tiny but strong features;^5^ crabs, lobsters, and sea urchin spines take advantage of ACC to stiffen and strengthen their skeletons.^6−9^ Such biomineralized designs provide great promising inspiration for constructing advanced bioenabled, lightweight structural materials with desired tailored functionalities.

To date, efforts have been made to mimic these natural ACC designs for manufacturing strong and tough functional mineralized composites with diverse applications in drug delivery,^8^ touch sensors,^10^ humidity actuators,^11^ battery binder,^12^ and microlens array.^13^ ACC possesses ultrasmall size down to 2 nm^14^ and polymer chain-like flexibility, leading to highly moldable complex morphologies as well as good transparency.^15,16^ However, the grand challenge facing the construction of ACC materials is their thermodynamically unstable behavior in the case of anhydrous ACC even transient nature under wet conditions.^5^ Synthetic ACC is inherently unstable within hours at room temperature and will rapidly convert to stable crystalline CaCO_3_ polymorphs such as calcite and aragonite.^5^ In contrast, naturally occurring ACC in living organisms is fairly stable in ambient environment, even maintaining good stability after being extracted from organisms.^17^ Such outstanding stabilization has been demonstrated to be associated with incorporated inorganic ions (e.g., magnesium ions, phosphate ions, silicate ions) and acidic organic biomacromolecules (e.g., polyaspartic acid-rich proteins, Asp-rich proteins).^4,18^ Thus, the above ions or polycarboxylates are widely used to extend the lifetime of synthetic ACC as the most conventional strategies. Among these, highly carboxylated species have been extensively explored to prevent ACC from nucleation and crystallization due to their high efficiency in calcium binding. However, these organic additives are usually either low efficient, expensive, poorly biocompatible, or nondegradable, limiting their potential application in specific fields, such as biodegradable materials, pharmaceuticals, and food packaging. It is urgent to explore “green” stabilizing agents that are highly efficient, biodegradable, biocompatible, sustainable, and low cost.

In crustacean cuticle, acidic Asp-rich proteins are decorated around rigid chitin nanofibers to stabilize ACC,^19^ which inspires high-efficient stabilizing agents design—the combination of chemical and geometric effects. Nanocellulose is the most abundant one-dimensional (1D) biopolymer produced from plants, bacteria, and tunicates.^20^ The parallelly packed macromolecular chains enable nanocellulose outstanding mechanical robustness with stiffness up to 150 GPa, even higher than chitin.^21^ Nanocellulose has been used to reinforce preformed and stabilized ACC materials for constructing stiff and transparent biomimetic composites.^22,23^ In these previous studies, the ACC nanoparticles were stabilized by extra stabilizing agents, including ethanol^22^ and poly(acrylic acid) (PAA),^23^ but they did not study the stabilization ability of nanocellulose itself. Hence, taking advantage of the rigid 1D geometry and surface chemistry of nanocellulose to extend the lifetime of ACC without additional stabilization agents needs systematic exploration. A fundamental understanding of whether and how the intrinsic properties of nanocellulose affect ACC composition, structure, and lifetime remains unknown.

Herein, we report the exceptional capability of cellulose nanofibrils (CNFs) for stabilizing ACC dispersions owing to their synergistic effect of rigid physically segregated networks and chemical complexation of calcium ions by carboxylic acid groups. The strong interfacial interaction allows calcium ions that are anchored on the CNFs surface to initiate the growth and stabilization of ACC to form a core–shell-like structure. The stabilization ability of CNFs depends linearly on the amount of carboxylic acid groups, and the stabilization efficiency of the highest carboxylated nanocellulose is 3.6 and 4.4 times higher than that of carboxymethyl cellulose (CMC) and alginate, although they have a similar carboxylated polysaccharide chemical structure. The stabilization efficiency is even better than the synthetic stabilizing agent and scale inhibitor – PAA.^24^ The CNFs also enable ACC dispersions with long-term stability for at least one month, which has been rarely achieved for synthetic ACC to date. Furthermore, the CNF/ACC dispersions could be further constructed into sustainable and transparent biomimetic composites with high strength, high toughness, self-healing, and humidity sensing ability. This outstanding combination of structural performance, multifunctionality, and sustainability facilitates various promising applications in plastic substitute structural materials, biomedicine, tissue engineering, and wearable devices.

## Results and Discussion

**Figure 1:**
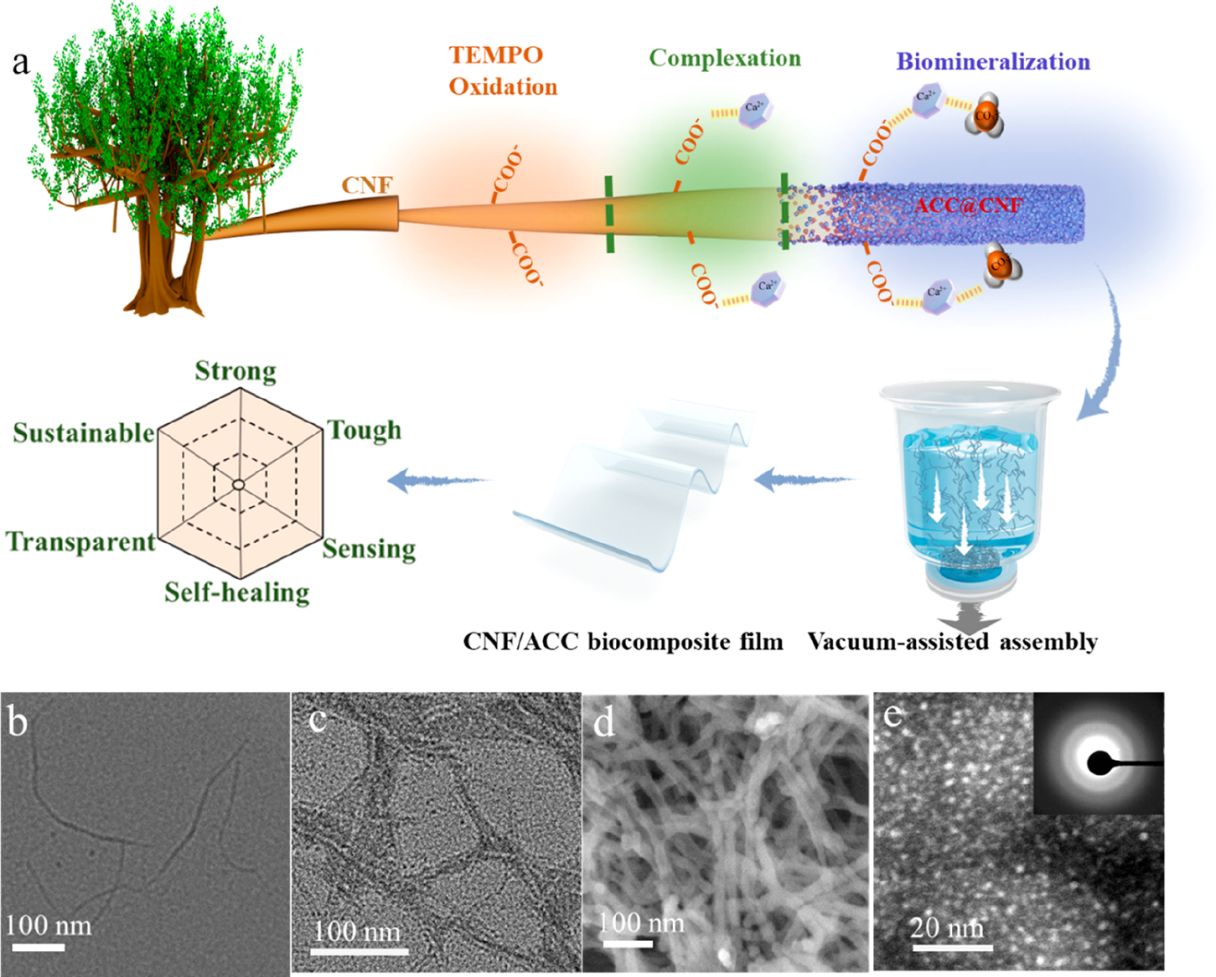
(a) Schematic illustration of the ACC stabilized by CNFs, which enables the construction of sustainable, strong, tough, transparent, self-healing, and sensing biocomposites. TEM images of (b) CNFs (T-2) and (c) CNF(T-2)/ACC. (d) SEM image of CNF(T-2)/ACC. (e) STEM image of ACC; inset: the corresponding SAED pattern.

Figure 1a illustrates the stabilizing process of ACC by carboxylated CNFs and the subsequent biocomposite films fabrication process. CNFs with a diameter of around 3–5 nm were extracted from softwood pulp by 2,2,6,6-tetramethylpiperidine-1-oxyl radical (TEMPO) oxidation twice (T-2),^25^ which caused the oxidation of a large number of hydroxyl groups of CNFs surface molecular chains to negatively charged carboxyl groups (Figure 1b). CaCl_2_ solution is subsequently added to the CNFs suspension, and the abundant active carboxyl groups of CNFs can capture free calcium ions for complexation to initiate the nucleation of ACC. Then, Na_2_CO_3_ solution in an equal amount of CaCl_2_ is added into the complexation system to provide CO_3_^2–^ binding with the CNFs-Ca^2+^. As a result, the ACC nanoparticles would grow and wrap around the CNFs surface to form a core–shell-like structure (Figure 1c). As shown in Figure 1d, the uniform fibril structure of ACC/CNF could be visualized clearly with a smooth surface, in sharp contrast to the rough aggregates of conventional PAA stabilized ACC (Figure S1). The strong calcium element signal of the corresponding energy dispersive X-ray (EDX) spectrum indicates the presence of calcium carbonate on the CNF surface (Figure S2). To further characterize the distribution and crystalline structure of the resulting ACC, we conducted additional scanning transmission electron microscopy (STEM) characterization, which exhibited continuous ultrasmall ACC clusters (1–2 nm) grown on the surface of CNFs (Figures 1e and S3), similar to the ultrasmall ACC nanoparticles in a PAA/ACC composite material.^16^ The amorphous character of these nanoclusters is confirmed by the appearance of diffuse rings in the selected area electron diffraction (SAED) pattern (inset Figure 1e). The resulting CNF/ACC suspension is highly transparent and stable for at least one month due to the outstanding templating and stabilization ability of CNFs (Figure S4), whereas CaCO_3_ suspensions synthesized by the same approach yet without CNFs exhibit turbidity caused by aggregates (Figure S5). The as-prepared CNF/ACC dispersions could be further constructed into sustainable and transparent biomimetic composites through vacuum assisted assembly technique, exhibiting high strength, high toughness, self-healing, and humidity sensing ability.

**Figure 2:**
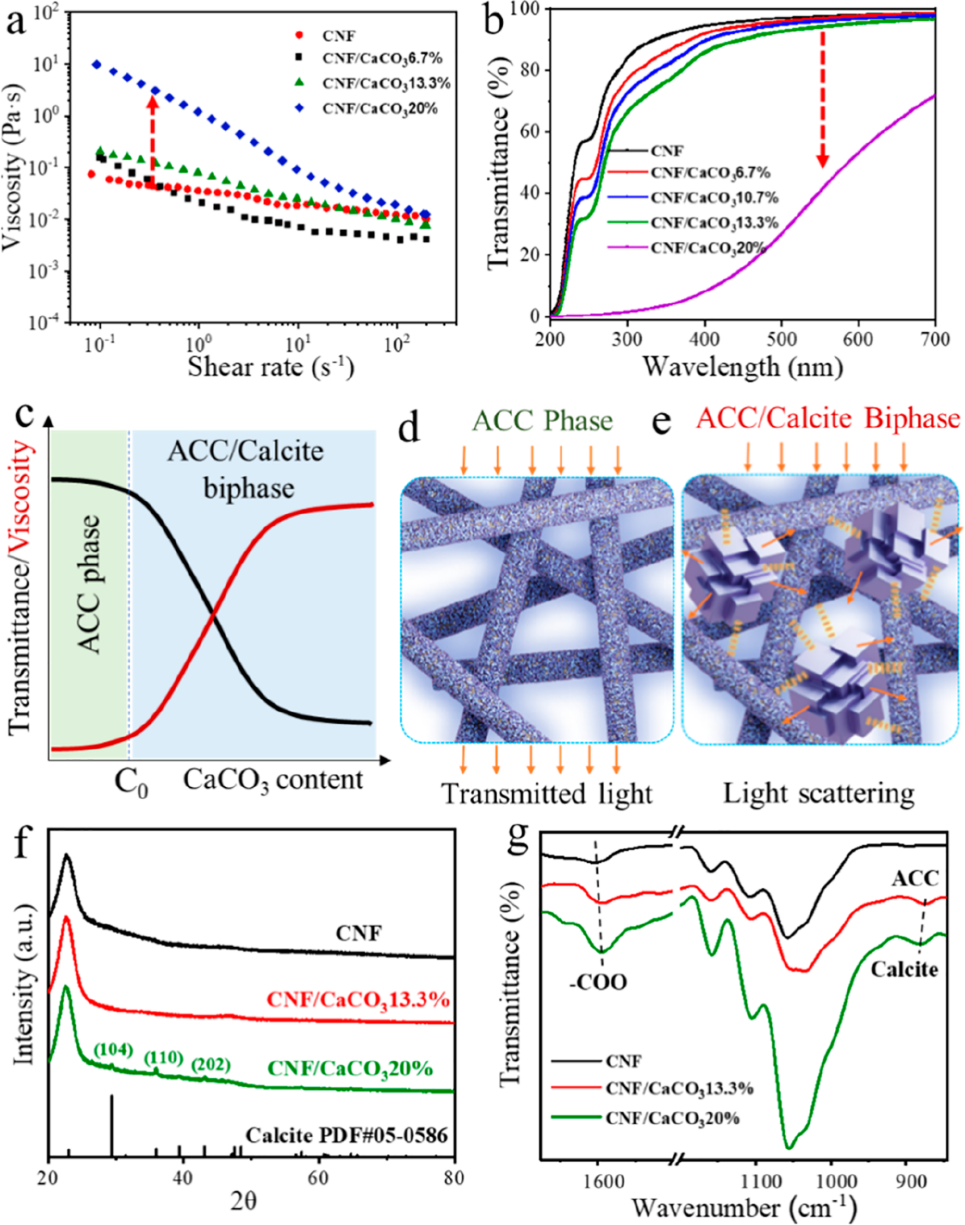
(a) Rheological behavior and (b) UV–vis spectra of CNF (T-2) and CNF/CaCO_3_ dispersions with different CaCO_3_ content. (c) Schematic illustration of transmittance and viscosity evolution behavior of CNF/CaCO_3_ suspensions with increasing CaCO_3_ content, where *C*_0_ denotes the onset of calcite crystallization. (d, e) Scheme of the nanostructure in ACC phase and ACC/Calcite biphase. (f) XRD patterns and (g) FTIR spectra of CNF, CNF/CaCO_3_ 13.3%, and CNF/CaCO_3_ 20%.

The effect of CNFs on ACC formation and crystallization in dispersions was monitored via the viscosity and transmittance changes of hydrid suspension, which are relevant to the particle interfacial interaction and size distribution.^26,27^ As shown in Figure 2a, the viscosity of CNF/CaCO_3_ suspensions declines as the shear rate increases, displaying a typical shear-thinning behavior due to the physical disentanglement of nanofibrils under shearing force. Interestingly, when the CaCO_3_ content is below a critical value of ca. 13 wt %, the rheological behavior of hybrid suspensions basically remains very similar. In contrast, further increasing the CaCO_3_ amount slightly above the critical content leads to a rapid viscosity increase by up to one order and more. A similar phenomenon could also be observed in the transmittance evolution of CNF/CaCO_3_ suspensions. The transmittance dramatically decreases from 95% to 70% at 550 nm when increasing the CaCO_3_ amount slightly above the critical content of ca. 13 wt % (Figure 2b), as the appearance of the suspension transforms from almost transparent to turbid (Figure S6).

We propose that this critical mineral content-dependent behavior is highly associated with the transition of ACC to crystalline calcium carbonates (Figure 2c). Below the critical mineral content, CaCO_3_ is in the single ACC phase that conformally wraps around CNFs, which leads to limited interaction with adjacent CNFs because of the rigid morphology of CNFs (Figure 2d). Thus, the rheology and transmittance are basically slightly affected by mineral content. By contrast, above the critical mineral content, the CaCO_3_ amount exceeds the maximum stabilizing capability of CNFs for ACC. The untemplated CaCO_3_ would aggregate and transition into large crystalline CaCO_3_ (Figure S7), which locates between adjacent CNFs to act as bridging sites (Figure 2e). These large aggregates would cause strong light scattering, resulting in a sharp decrease of transmittance, while the enhanced interactions could increase the viscosity, as proven by the gelation of a hybrid suspension above the critical mineral content (Figure S8). To verify this proposed mechanism, we carried out X-ray diffraction (XRD) and Fourier-transform infrared (FTIR) spectroscopy. The XRD spectra exhibit that the CNF/CaCO_3_ sample with minerals above the critical content indeed changes from ACC to crystalline calcium carbonate (Figure 2f), whose diffraction peaks attribute to calcite (PDF#05-0586). Besides, the absorption peak at 875 cm^–1^ of the FTIR spectrum corresponding to ACC also shifts to 880 cm^–1^, the characteristic peak of calcite (Figure 2g), further confirming calcite growth in the hybrid suspension above the critical mineral content. These results suggest that the critical mineral content (*C*_0_) is the maximum stabilizing capability of CNFs for ACC.

**Figure 3:**
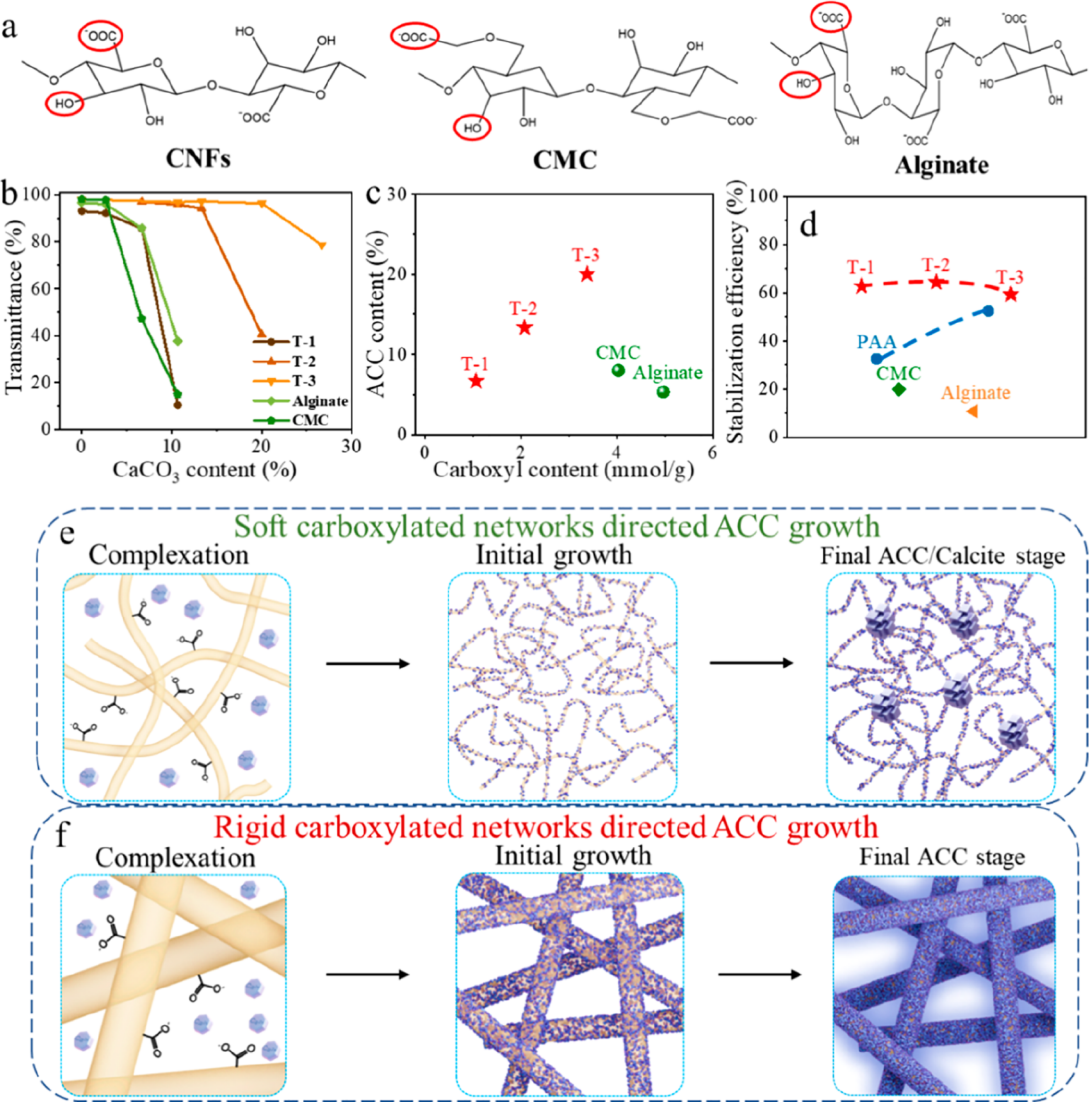
(a) Molecular formula of CNF, CMC, and alginate. (b) Transmittance changes of T-1/CaCO_3_, T-2/CaCO_3_, T-3/CaCO_3_, CMC/CaCO_3_, and alginate/CaCO_3_ with different CaCO_3_ content. (c) The maximum stabilized amount of ACC enabled by T-1, T-2, T-3, CMC, and alginate. (d) Stabilization efficiency of the carboxyl groups in different carboxylated polymers. Schematic diagram of the growth process of ACC induced by soft carboxylated polymer chains networks (e) and rigid carboxylated CNF networks (f).

To gain insight into how the surface carboxyl groups and physical rigid nanofibril network affect the stabilization of ACC, a series of CNFs with different amounts of carboxyl groups were fabricated by different TEMPO oxidization times, which are marked as T-1, T-2, and T-3 for TEMPO application once, twice, and three times, respectively. The resulting carboxyl group content of T-1, T-2, and T-3 was measured by conductometric titration to be 1.06, 2.07, and 3.38 mmol/g, respectively (Figure S9a–c). The increased surface carboxyl groups lead to the decrease of dispersion viscosity due to the enhanced electrostatic repulsive force in the neutral pH (Figure S10a). Besides, these CNFs share a similar viscosity and transmittance transition phenomenon, despite they contain different amounts of surface carboxyl groups (Figures S10b–d and S11). Additionally, two other biopolymers, CMC and sodium alginate, are also utilized to stabilize ACC for comparison due to their similar chemical structure to CNFs (Figure 3a). The carboxylate group content was determined to be 4.09 and 4.97 mmol/g for CMC and alginate, respectively (Figure S9d,e), which are close to the theoretical values calculated from the chemical formulas. The maximum stabilizing capability of CNFs, CMC, and alginate for ACC was determined by the transmittance transition and XRD characterization according to the prediscussed methods (Figures 3b, S11, and S12). It can be found that the stabilized ACC content almost increases linearly with the increase of carboxyl group amount on the CNFs surface, and the maximum capacity for stabilizing ACC is around 20 wt % (Figure 3c). This result indicates that the carboxyl groups play a critical role in stabilizing ACC. However, although CMC and alginate have a higher carboxylate content than CNFs, their stabilized ACC content is only 0.25 and 0.4 times of T-3 stabilized ACC, respectively. The stabilization efficiency of the carboxyl group for ACC is calculated according to the following equation:
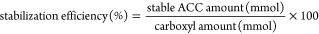


Notably, the stabilization efficiency of carboxyl groups on the CNFs surface is calculated to be in the range of 59.2%–64.3% (Figure 3d). In contrast, CMC and alginate exhibited much lower efficiency with only 19.9% and 10.8%, respectively. This value indicates that each carboxyl group of CNFs can stabilize more than the stoichiometric 50% expected for CaCO_3_ (COO^–^-Ca^2+^-COO^–^ complex). This outstanding stabilization efficiency is even higher than the most used stabilizing agent, PAA, which exhibits an efficiency of 32.5%–52.5% (*M*_w_ = 5000)^24^ and also higher than that of specially designed triblock copolymers for simultaneous Ca^2+^ and cholesterol binding (5–30%).^28^

Although all the molecular structural formulas of all three biopolymers (CNFs, CMC, and alginate) contain hydroxyl and carboxyl groups, they differ significantly in their ability to stabilize ACC. We suggest that this exceptional stabilization capability of CNFs for ACC is related to the synergistic effect of the carboxyl group chemical structure and the physically rigid fibrillar networks. CMC and alginate possess soft and long molecular chains that could transform into coiled shrinking conformations induced by the strong coordination of the carboxylate group and Ca^2+^ (Figure 3e). The dense conformations facilitate the ordered coordination of Ca^2+^ with the flexible biopolymer molecular chains for nucleation and growth of crystalline CaCO_3_.^24^ In contrast, CNFs possess ultrarigid nanofibril networks with abundant carboxylate surfaces, which can act as the nanotemplate and physically segregated structure for Ca^2+^ coordinating along the nanofibrils (Figure 3f). These anchored Ca^2+^ have limited freedom to interact with each other to form an ordered arrangement, thus inhibiting the crystalline CaCO_3_ nucleation. Other studies have also reported the physical confinement effect on the stabilization of ACC.^29,30^ A similar stabilization mechanism has also been used to explain the molecular-weight dependent stabilization behavior of PAA for ACC, where the rigid stretched conformation of the shorter chained PAA is more favorable for ACC stabilization than the coiled conformation of long chains.^24^

**Figure 4:**
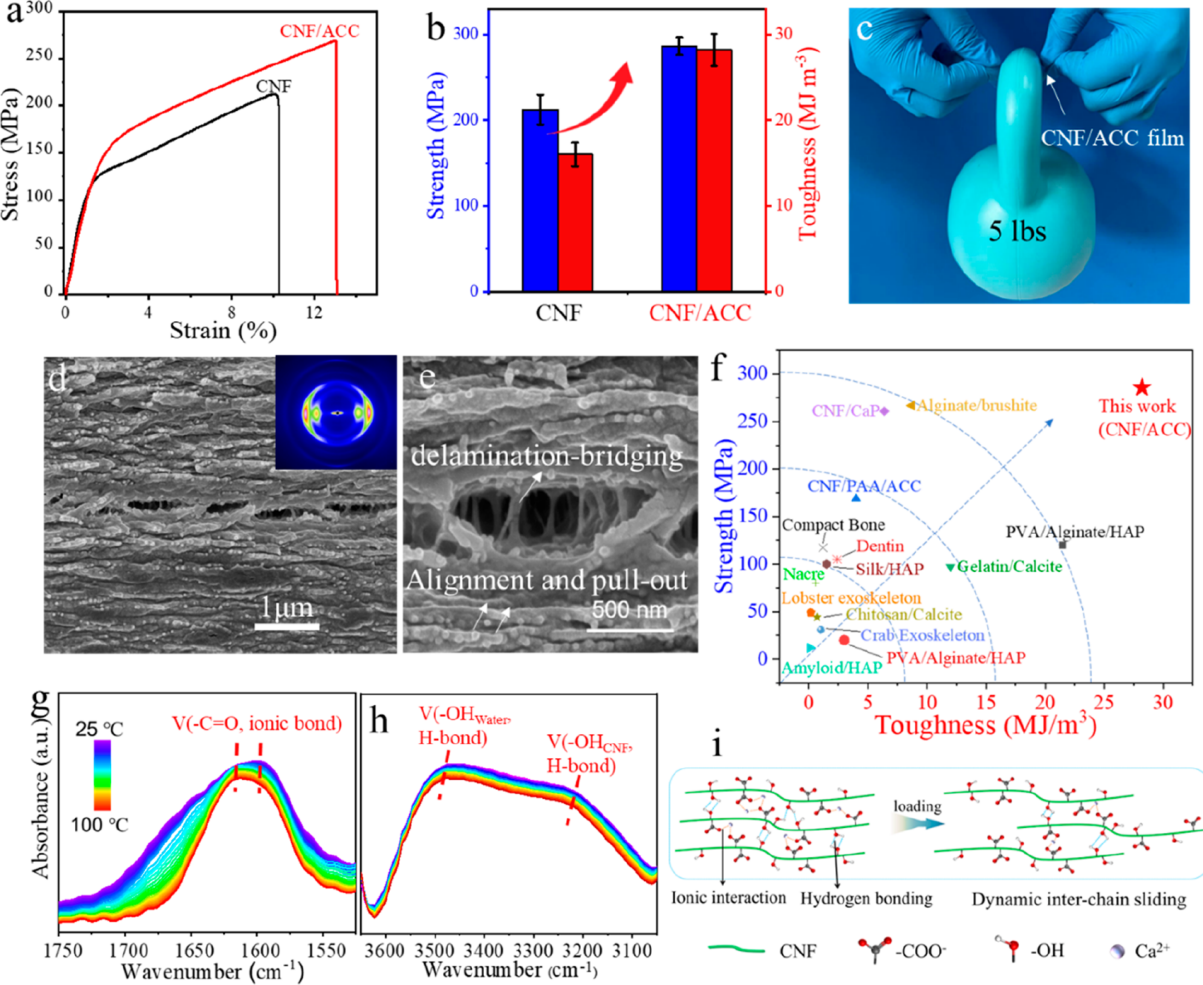
(a) Typical stress–strain curves of CNF film and CNF/ACC composite film. (b) Strength and toughness comparison of CNF film and CNF/ACC composite film. (c) Photo of the CNF/ACC composite film lifting a weight of 5 lbs. (d, e) SEM of the fractured cross-section of CNF/ACC films; inset of (d): the corresponding 2D XRD pattern. (f) Mechanical comparison of our CNF/ACC composite film with natural mineralized composites and artificial biopolymer-calcium carbonate/phosphate composites. (g, h) The temperature-dependent FTIR spectrum of the CNF/ACC composite film. (i) The schematic of adaptive dynamic interfacial bonding upon the force loading.

Generally, high strength and toughness are mutually exclusive in synthetic composites due to their conflicting localized reinforcement and deformation mechanisms.^31^ However, the as-prepared CNF/ACC hybrid suspensions can be assembled into transparent, strong, and tough composite films through vacuum-assisted assembly techniques.^32^ The CNFs (T-1) were selected to construct composite films due to their relatively simpler extraction procedure than T-2 or T-3 for large-scale preparation. The as-prepared CNF/ACC film is approximately 30 μm thick and shows high transparency in the visible spectrum with over 90% transmittance, which is even slightly higher than that of a CNF film (Figure S13). The enhanced transparency might be related to the possibility that the ultrasmall ACC nanoparticles can fill up the voids of CNF films to obtain more uniform and dense hybrid nanostructures with a more uniform refractive index as compared to the CNF films. The stress–strain curves of CNF and CNF/ACC films exhibit a typical plastic behavior due to the interfibrillar debonding and subsequent fibrillar sliding of the nanofibrillated structure (Figure 4a).^33^ The corporation of ACC with CNF is able to significantly enhance the mechanical properties and achieve an exceptional combination of high strength of 286 ± 10 MPa (up to 300 MPa in some cases) and high toughness of 28.2 ± 1.9 MJ/m^3^ (Figures 4b and S14).^34^ This as-prepared film (3 cm × 7 cm) even is able to lift a weight of 5 lbs. To gain a comprehensive overview of the resulting mechanical properties, we compared the strength and toughness of the reported biopolymer-calcium carbonate/phosphate composites and natural mineralized composites (e.g., crustacean cuticles, bone, and dentin). As shown in Figure 4f (Table S1), the strength and toughness of natural mineralized composites are usually in the range of 30–120 MPa and 0.2–2.4 MJ/m^3^, respectively.^34−38^ In contrast, artificial mineralized materials have been constructed with enhanced mechanical performance. Notably, our CNF/ACC composites extend beyond the property space of these reported mineralized materials.^23,39−46^ For example, the composite films composed of CNF and PAA stabilized ACC exhibit a strength of 169 MPa and toughness of around 4 MJ/m^3^, which is far weaker than our composites film, despite the similar building blocks.^23^ The in situ growth of CaP into the CNF matrix resulted in high strength of 261 MPa close to our study, but the toughness is modest with around 6.4 MJ/m^3^.^42^

These outstanding mechanical properties are suggested to be related to the hierarchical structural deformation of laminated structures and favorable interfacial interactions. The fractured cross-section of CNF/ACC films exhibits a laminated structure accompanied by the in-plane two-dimensional X-ray diffraction (2D XRD) pattern with an orientational degree of 0.67 (Figures 4d and S15), which stems from the layer-by-layer self-assembly of CNF/ACC nanofibrils and densification during drying. Closer observation reveals the pull-out of aligned nanofibrils from layers as well as rough delaminated morphology that is bridged by nanofibrils (Figure 4e). The hierarchical deformation could significantly benefit from the energy dissipation for achieving exceptionally high mechanical characteristics. Additionally, the temperature-dependent FTIR is employed to analyze the interfacial interaction of the assembled CNF/ACC composite. While heating from 25 to 100 °C, the band intensity of the ionic-bonded C=O group between CNFs and ACC at 1590 cm^–1^ keeps decreasing and shifting to 1612 cm^–1^ during the whole heating process, indicating the strong ionic interaction between CNFs and ACC (Figure 4g). Meanwhile, the stretching vibration bands near 3210 cm^–1^ belong to the hydroxyl groups of CNFs, which gradually decrease and shift to 3222 cm^–1^ (Figure 4h), also suggesting the presence of strong hydrogen bonds between adjacent CNFs. Upon the force loading, the dynamic interfacial interaction, including ionic bonds and hydrogen bonds, would adaptively break and reform during the CNF sliding and pull-out events, which significantly facilitates the efficient loading transfer for the mechanical robustness (Figure 4i).

**Figure 5:**
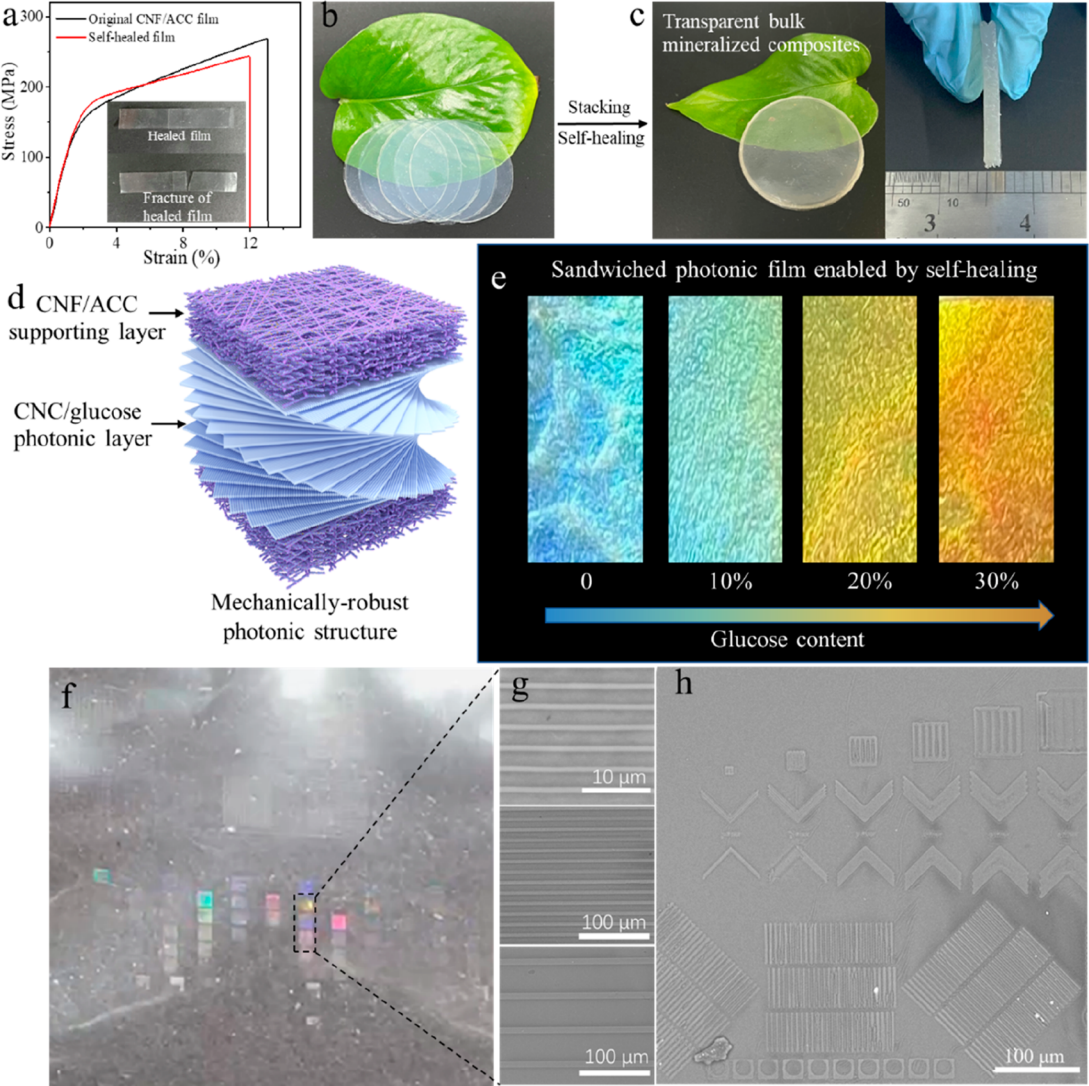
(a) Typical stress–strain curves of the origin CNF/ACC film and self-healed film; inset: photos of the healed film and the fracture of the healed film. (b, c) Photos of transparent bulk mineralized composites by stacking and self-healing of many pieces of CNF/ACC films. (d) Schematic illustration of the strong sandwiched photonic film consisting of two CNF/ACC supporting layers and a CNC photonic layer. (e) Photos of strong and flexible sandwiched films with different structural color enabled by different amount addition of glucose. (f) Photo of CNF/ACC film with structural color because of imprinted surface grating structure. (g) SEM images of imprinted grating structure with different periodicities. (h) SEM images of other imprinted structures with different geometries and sizes.

Besides, the strong interfacial strength also enables the CNF/ACC films to have good self-healing properties. By attaching two pieces of hydrated films together, the hydrogen bonding and strong interfacial electrostatic interaction between COO^–^ and Ca^2+^ similar to CaCO_3_/PAA composite materials could firmly bond them to form a stable freestanding film.^16^ The stress–strain curves indicate that there is no significant decrease in mechanical properties after self-healing (Figure 5a). The mechanical healing efficiency (η), which is defined as the recovery of toughness, was also investigated. Our composite shows a good η of 88.35% at room temperature.^47^ The resulting tensile fracture usually took place outside the healed region, also revealing the good healing of the CNF/ACC film (inset in Figure 5a). Stacking many pieces of films together, we could construct 1.5 mm-thick transparent bulk mineralized materials that are rarely achieved in mineralized composites (Figure 5b,c).^15^

Cellulose nanocrystals (CNCs) are able to self-assemble into a solid chiral nematic liquid crystal structure with bright iridescence. However, these CNC photonic films are extremely brittle and weak, which significantly limits their practical applications.^48^ By taking advantage of the self-healing property of our composite film, we could sandwich a chiral nematic cellulose nanocrystal (CNC) film between two pieces of CNF/ACC films to construct a nanocellulose photonic film with good strength and flexibility (Figure 5d). The high transparency of the CNF/ACC film allows good light transmittance to interact with the inner chiral nematic for brilliant structural iridescence. By incorporation of glucose into the CNC chiral nematic matrix to tailor the photonic band, different structural colors expanding over the visible light wavelength can be obtained (Figures 5e and S16). With the increasing addition of glucose, the light reflection peak systematically shifts to the red. The increased photonic bands are caused by the seamless intercalation of glucose into the original CNC helicoidal organization via interstitial volumes within nanocrystals and between nematic monolayers.^49^ Additionally, the mechanical robustness of the CNF/ACC film can provide mechanical support to largely improve the mechanical properties of the CNC photonic film (Figure S17), which can address the challenge that the mechanical brittleness of CNC films.

Additionally, owing to the good mechanical properties, our CNF/ACC films can fabricate surface patterns with high resolution down to submicrometer using imprint technique, which is in analogy to natural ACC that can be molded into various complex geometries by living creatures (Figure 5f). For instance, diverse grating structures with different periodicities were integrated on the film surface (Figure 5g). The resulting large-area diffraction grating patterns reveal vivid dynamic structural color when tunning the view angle (Figure 5f, Video S1). By changing the predesigned molds, physical patterns with diverse geometries and feature size ranging from cm- to nm-level could be perfectly replicated onto the PAA/ACC surface (Figure 5h), which is very attractive for potential surface functionalities.

**Figure 6:**
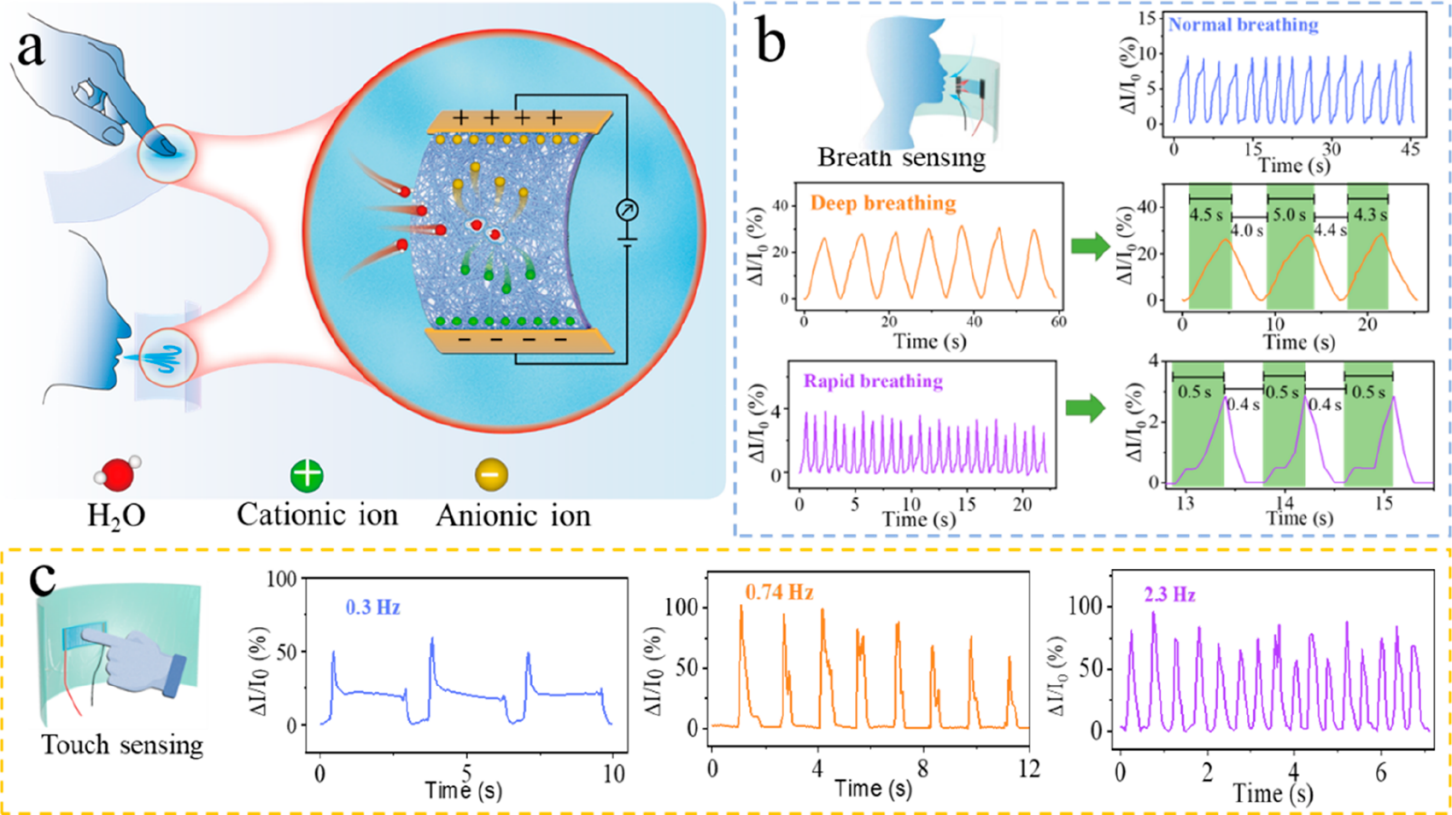
(a) Schematic illustration of the CNF/ACC composite humidity sensor for monitoring respiration and finger contact. (b) Current change rates (Δ*I*/*I*_0_) of three respiration patterns monitored by the CNF/ACC composite film humidity sensor: normal breathing, deep breathing, and rapid breathing. (c) Current change rates (Δ*I*/*I*_0_) of three fingertip contact patterns monitored by the CNF/ACC composite film humidity sensor: 0.3, 0.74, and 2.3 Hz.

Finally, in sharp contrast to the unstable behavior of CNF films in a wet environment (Figure S18), the CNF/ACC film can maintain good wet mechanical integrity even under intense ultrasonication owing to the strong ionic interaction between CNF and ACC. This wet stability combined with the slightly dissolved free Ca^2+^ from ACC serving as the ion carriers is attractive for humidity sensing. To enhance the sensing signal, additional NaCl is introduced to serve as the sensitive element by immersing the composite film in saturated NaCl aqueous solution followed by drying. Figure 6a shows the schematic illustration of the humidity sensor for monitoring respiration and finger contact with the CNF/ACC composite. The current change rates (Δ*I*/*I*_0_) are used to monitor different breathing and fingertip contact states by the CNF/ACC composite film humidity sensor. The resulting sensor is capable of distinguishing the variation of the breathing rate and fingertip contact frequency with a good response/recovery sensitivity, i.e., normal breathing, rapid breathing, and deep breathing (Figure 6b) as well as different frequency of 0.3 Hz, 2.3 Hz, and 0.74 Hz (Figure 6c).

The excellent moisture sensitivity of our CNF/ACC composites is due to their hierarchical molecular structure. The abundant hydroxyl groups and amorphous minerals significantly facilitate the attraction of water molecules. During the diffusion of water molecules, hydrogen bonding and ionic interactions are formed with the cellulose substrate in the composite film, resulting in a change in the dielectric constant or resistance of the material, which enables the sensing of humidity and the monitoring of changes in humidity.^50^ Accordingly, the obtained CNF/ACC composite film shows high sensitivity and stability, small hysteresis, and rapid response/recovery time to humidity. Therefore, it can be used to assemble skin moisture detectors with high flexibility and transparency, which can offer real-time moisture information for intelligent wearable devices.

## Conclusion

Inspired by the combination of acidic Asp-rich proteins and chitin nanofibers to stabilize ACC in natural crustacean cuticles, we report that carboxylated CNFs have an exceptional ability to template and stabilize ACC. The underlying stabilizing ability stems from the synergistic effect of abundant carboxyl groups and rigid nanofibril geometry. Carboxyl groups are in charge of complexing Ca^2+^/ACC, while rigid geometry is further responsible for limiting the freedom of ACC, thus inhibiting mineral crystallization. The resulting stabilization efficiency is well beyond carboxylated biopolymers (CMC and alginate) and synthetic PAA (*M*_w_ = 5000 g/mol). This success of combining chemical and physical stabilizing mechanisms into single biopolymer materials has important reference value for designing other stabilizing agents of amorphous minerals. Additionally, taking advantage of this CNF/ACC hybrid, we could construct transparent composites with an outstanding combination of strength and toughness that exceed these reported biopolymer-calcium carbonate/phosphate materials. The as-prepared composite films also exhibit self-healing and wet-stability that have been used to fabricate humidity sensors for monitoring respiration and finger contact with good sensitivity. The sustainable and biological basis, efficiency, and versatile functionalities (e.g., mechanically robust features, transparency, self-healing, patternable) of our composites make them attractive for multiple applications in biomedicine, packaging, and wearable devices.

## Experimental Section

### Materials

Bleached wood pulp was purchased from Dalian Yangrun Trading Co., Ltd., China. 2,2,6,6-Tetramethylpiperidine-1-oxyl (TEMPO), sodium hydroxide (NaOH), anhydrous calcium chloride (CaCl_2_), sodium carbonate (Na_2_CO_3_), sodium alginate (SA), and carboxymethyl cellulose (CMC) were purchased from Aladdin Industrial Co., Shanghai, China. Sulfuric acid, sodium bromide (NaBr), sodium hypochlorite (NaClO), and hydrochloric acid were purchased from Xilong Scientific Co., Ltd., China.

### Preparation of CNFs

The CNF was extracted from wood pulp according to our previously reported study.^51^ Briefly, bleached wood pulp was pulverized by a high-speed multifunctional crusher. The obtained cellulose powder (30 g) was added to distilled water (3000 mL). Then, a pH meter was placed in the suspension to monitor its pH value. After being vigorously stirred for several hours, TEMPO (0.468 g), NaBr (3.086 g), and 11 wt % NaClO (193.6 g) were separately added in the above suspension. Under constant stirring, the 0.1 M HCl was used to adjust the pH of the system to maintain it at around 10. As the reaction proceeds, the pH of the solution decreases. To maintain the pH of the system, 0.5 M NaOH solution was added slowly. The reaction was terminated when there is no change of the pH in the system for at least 30 min. The as-prepared TEMPO-oxidized cellulose slurry was thoroughly washed to neutral and diluted to 0.5 wt % cellulose fiber suspension. The CNFs were extracted from the above suspension by ultrasonication (JY99-IICN, Ningbo Scientz Biotechnology Co., Ltd., China). In the end, the CNF suspensions were centrifuged (TG16-WS, Xiangyi, Changsha, China) at 10,000 rpm for 30 min to remove the undispersed impurities, which were named T-1. The above-oxidized cellulose slurry was reoxidized, washed, and repeated the sonication and centrifugation operations, named T-2. The above twice-oxidized cellulose slurry was reoxidized, washed, and repeated the sonication and centrifugation operations, named T-3.

### Stabilization of ACC by CNFs Suspensions

In a typical process, CaCl_2_ solution (0.2 M) was added to the CNF suspension (T-2, 0.3 wt %). After being vigorously stirred for 30 min, Na_2_CO_3_ solution (0.2 M) in an equal amount with CaCl_2_ was added to CNF/CaCl_2_ suspension. The reaction lasted for 30 min under constant stirring.

### Preparation of CNF/ACC Composite Films

The CNF/ACC composite film was fabricated by vacuum filtration of the mixed CNF/ACC suspension on a cellulose filter membrane with a 0.4 μm pore size to make sure that the CNF/ACC mixture was retained on the filtration membrane. The as-prepared composite films were left at room temperature to dry thoroughly in air and then peeled off from the cellulose filter membrane.

### Characterization

The morphologies of samples were observed using the FEI-INSPECTF scanning electron microscopy (SEM, Hillsboro, OR, USA). Samples were coated with gold at 15 mA for 3 min and then examined at 5.0 kV. Transmission electron microscopy (TEM) was used to reveal the morphologies of ACC nanoparticles at 120 kV. To study the effects of different TEMPO oxidization times and ACC contents on CNF suspensions, the rheological tests were performed by an advanced rheometer (AR2000EX, TA Instruments, USA). The strain was set at 10%, and the frequency ranged from 100 to 0.05 Hz. Fourier transform infrared (FTIR) spectroscopy (Magna-IR 560, USA) was performed in a wavenumber range of 4000–700 cm^–1^ with a resolution of 4 cm^–1^. A Nicolet iS50 Fourier transform spectrometer equipped with a deuterated triglycine sulfate detector was used for the temperature-dependent FTIR measurements. The sample was heated from 25 to 100 °C at 2 °C min^–1^, and the temperature-dependent FTIR spectra within the region from 4000 to 700 cm^–1^ were collected at the same time. A total of 38 FTIR spectra were collected upon heating. The crystalline structures of the crystallized calcium carbonate were recorded by X-ray diffractometer (XRD, Philip X’ Pert PRO MPD, The Netherlands). The wide-angle X-ray scattering (WAXS) data of the sample were collected on the WAXS beamline at the Nanopix (Rigaku Corporation, Japan). The radiation source is Cu Kα radiation (λ = 1.54 Å) with a 50 mA generator current and 40 kV generator voltage, and the scanning speed is 10° min^–1^. The 2θ angle ranges from 20° to 80°. The light transmittance of the samples was analyzed in the wavelength range of 300–700 nm by means of an Agilent Cary 60 UV–vis spectrometer (Agilent Technologies, Santa Clara, USA). Thermal gravimetric analyzer (TGA) measurement was recorded by TGA instrument (NETZSCH TG209F3, Germany) under nitrogen atmosphere at a heating rate of 10 °C min^–1^.

### Mechanical and Self-Healing Tests

Tensile stress–strain testing of the samples was measured by Instron 5567 universal testing machine (Norwood, MA, USA) with a 1 kN load cell. The mechanical and self-healing tests were performed at a strain rate of 1 mm min^–1^ at room temperature and humidity of 40–50%. There is a very small amount of Na atoms (around 0.61 wt %) in the composite materials (Figure S19), because Na ions are the counterions of the carboxy groups on CNFs during the CNF preparation process. The water molecule content in the composite is around 4.5 wt % according to TGA characterization (Figure S20). The samples were cut by length and width with 2 mm × 10 mm × *d* mm, where *d* was the thickness of samples. The sample’s thickness was measured using a digital micrometer (MC0300002, Guilin Guanglu Measuring Instrument Co., China). Damaged samples which were cut by a single edge blade into two halves were sprayed with water and then given 24 h at room temperature before tensile measurements. The result of tensile strength, elongation at break, and self-healing efficiency were determined from data of 5 random measurements of the sample.

## Supporting Information Available

Figures S1–S19: SEM images of PAA stabilized ACC; EDX spectrum of CNF/ACC film; size distribution of the CNFs stabilized ACC; photos of CNF/ACC dispersions at ambient environment for different times and the corresponding XRD patterns; photos of freshly prepared CaCO_3_ dispersion with and without CNFs; photos of transmittance change of CNF/CaCO_3_ dispersions with different CaCO_3_ content; TEM of CNF/CaCO_3_ 20%; photos of CNF/CaCO_3_ dispersion with 13.3% CaCO_3_ and 20% CaCO_3_; conductometric titration curves of T-1, T-2, T-3, CMC, and alginate; rheological behavior for T-1, T-2, T-3, and their CNF/CaCO_3_ dispersions with different CaCO_3_ contents; UV–vis spectra, XRD patterns and photos of T-1, T-2, T-3, CMC, and alginate with different CaCO_3_ contents; UV–vis spectra of CNF and CNF/ACC film; strength and toughness of CNF/ACC composite films with different CaCO_3_ content; TGA curve and azimuthal plot of the 2D XRD Pattern of the CNF/ACC composite film; UV–vis spectra of sandwiched photonic film with different amount of glucose loading; Stress–strain curves of CNC and sandwiched CNC film; photos of CNF film and CNF/ACC composite film in water for 0.5 h. Table S1: the mechanical properties of natural mineralized composites and reported biopolymer-calcium carbonate/phosphate composites (PDF)
Video S1: The dynamic structural color of CNF/ACC film enabled by the surface grating structure (MP4)
