# Supplementary material for: Bioinspired Stabilization of Amorphous Calcium Carbonate by Carboxylated Nanocellulose Enables Mechanically Robust, Healable, and Sensing Biocomposites
Source: ACS Nano. 2023 Mar 22;17(7):6664–74. doi: 10.1021/acsnano.2c12385 (PMC10100558; doi:10.1021/acsnano.2c12385)
Supplement: Supplementary file 1 — nn2c12385_si_001.pdf [file nn2c12385_si_001.pdf]

Supporting information

Bioinspired Stabilization of Amorphous Calcium Carbonate by Carboxylated Nanocellulose enables Mechanically-Robust, Healable, and Sensing Bio-Composites

Wanlin Wu[1#], Zhixing Lu[2#], Canhui Lu[1], Xunwen Sun[1], Bing Ni[3], Helmut Cölfen[3] & Rui Xiong[1*]*

[1] State Key Laboratory of Polymer Materials Engineering, Polymer Research Institute of Sichuan University, Chengdu, 610065, China

[2] Engineering Research Center of Polymer Green Recycling of Ministry of Education, College of Environmental and Resource Sciences, Fujian Normal University, Fuzhou, 350007, China

[3] Physical Chemistry, Department of Chemistry, University of Konstanz, Konstanz, 78457, Germany

* Corresponding author. E-mail addresses: helmut.coelfen@uni-konstanz.de (H. C.); rui.xiong@scu.edu.cn (R. X.)

#W.W. and Z.L. contribute equally to this work.

Supporting Figures

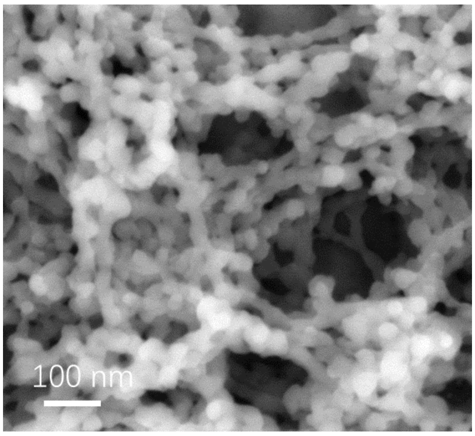

Figure S1. SEM images of PAA stabilized ACC products.

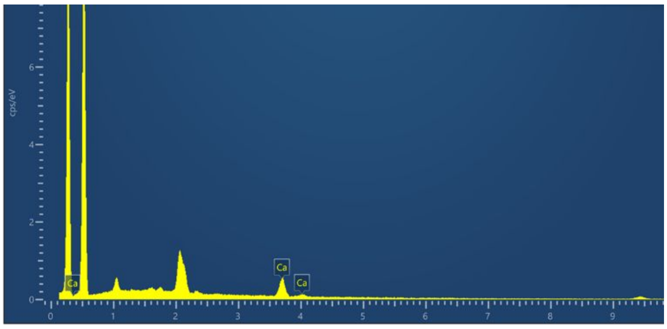

Figure S2. EDX spectrum of the as-prepared CNF/ACC film.

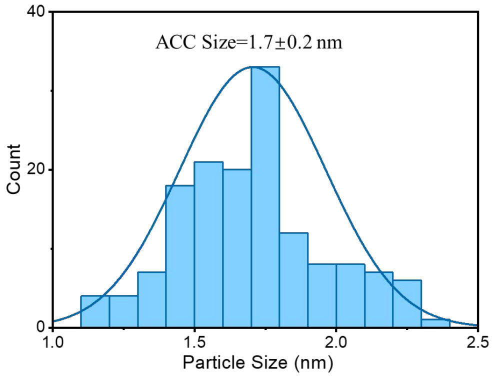

Figure S3. Size distribution of the CNFs stabilized ACC measured from TEM images.

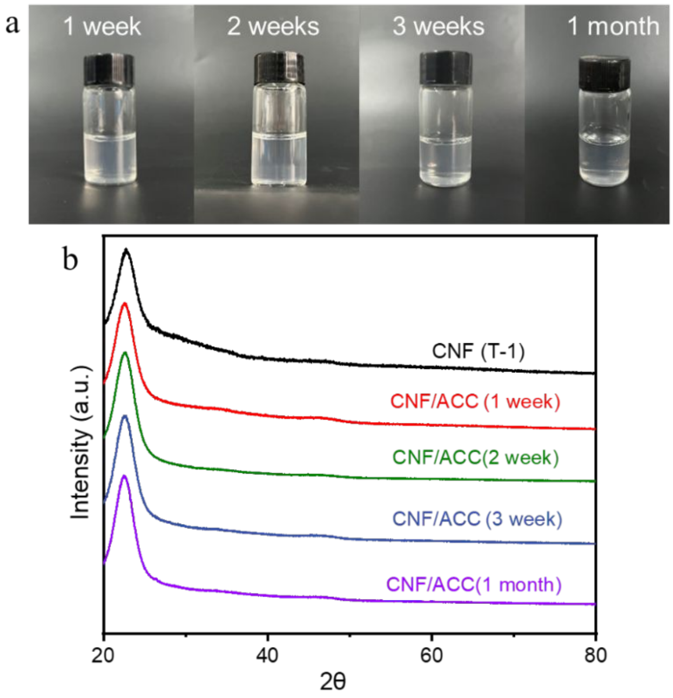

Figure S4. (a) Photos of CNF/ACC dispersions have been taken at ambient environment for different times; (b) The corresponding XRD patterns indicate the amorphous character of $CaCO_3$.

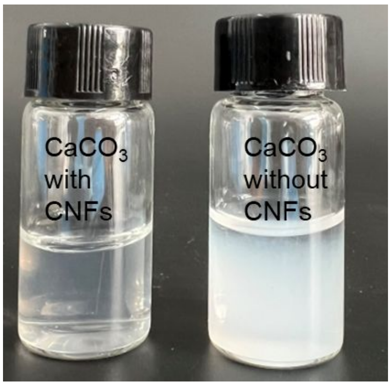

Figure S5. Photos of freshly-prepared $CaCO_3$ dispersion with and without CNFs.

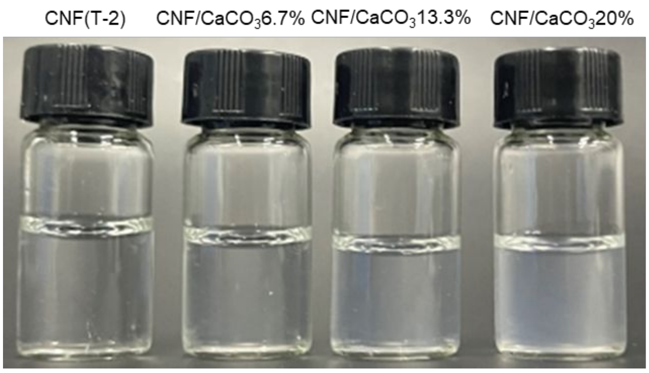

Figure S6. Photos of transmittance change of CNF/$CaCO_3$ dispersions with different $CaCO_3$ content.

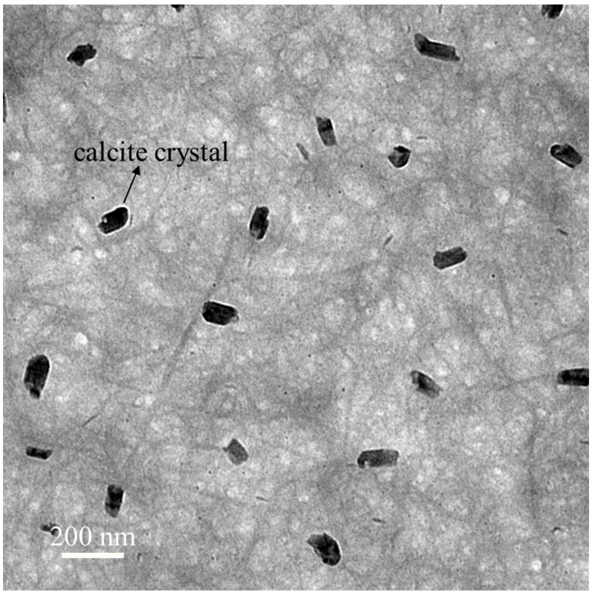

Figure S7. TEM of as-prepared CNF/$CaCO_3$ 20%, which exhibits numerous large calcite crystals existing in the CNF network.

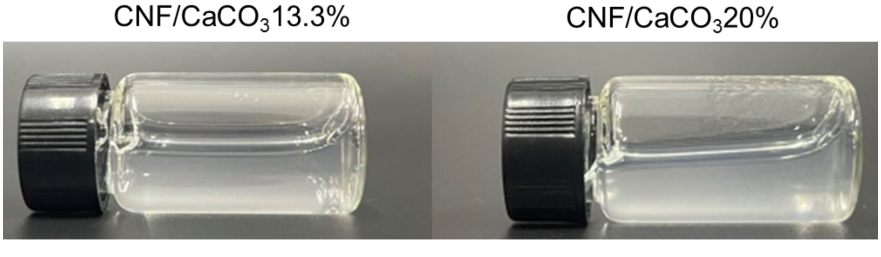

Figure S8. Photos of a CNF/$CaCO_3$ dispersion with 13.3% $CaCO_3$ and 20% $CaCO_3$,

where CNF/$CaCO_3$20% starts gelation due to the strong additional ionic crosslinking by the large calcite particles.

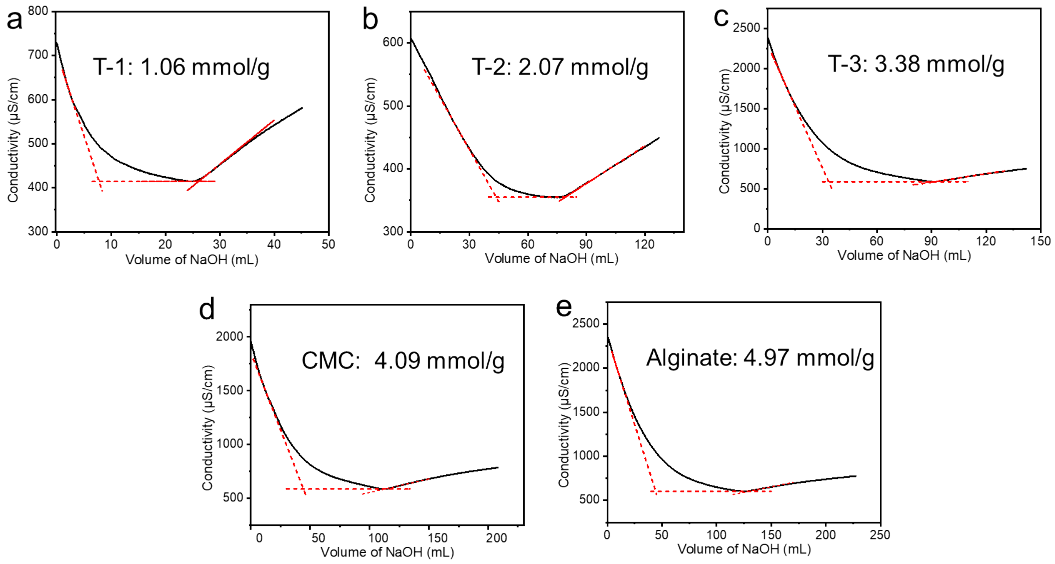

Figure S9. Conductometric titration curves of (a) T-1, (b) T-2, (c) T-3, (d) CMC, and (e) alginate for calculating the carboxyl group content.

The carboxyl group content of the suspensions was determined via conductometric titration.[1] In brief, 5 mL, 0.01 M NaCl solution was added into 40 mL, 0.3 wt.% suspension with constant stirring. The pH of the suspension was adjusted to about 2.5 by 0.1 M HCl. Then, 0.007 M NaOH solution was added into the suspension at the rate of 0.1 mL/min until the pH reached about 11. The volume of NaOH solution was recorded, and the corresponding conductivity change of the suspension was measured by the conductometer. The graph of conductometric titration of T-1, T-2, T-3, CMC, and Alginate is shown in Figure S1. The conductivity decreased until the acid was neutralized by the addition of NaOH solution (V_1). With the continuous addition of

NaOH solution, the conductivity remained unchanged (V_2). The carboxylate group content was determined by means of Eq., presented below:

$$\text{Carboxyl content}\left(\frac{mmol}{g}\right) = \frac{(V2 - V1) \times M\ NaOH}{weight\ of\ cellulose\ (CMC\ or\ Alginate)}$$

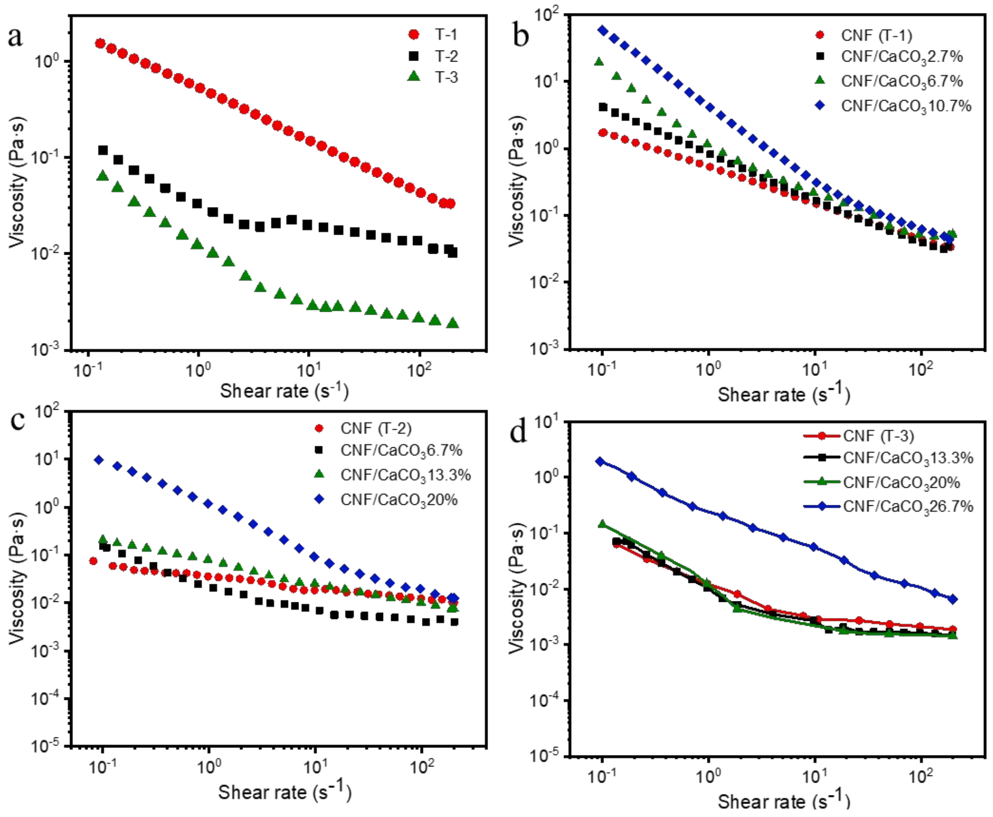

Figure S10. Rheological behavior for (a) T-1, T2, T3, and (b-d) their CNF/$CaCO_3$ dispersions with different $CaCO_3$ contents.

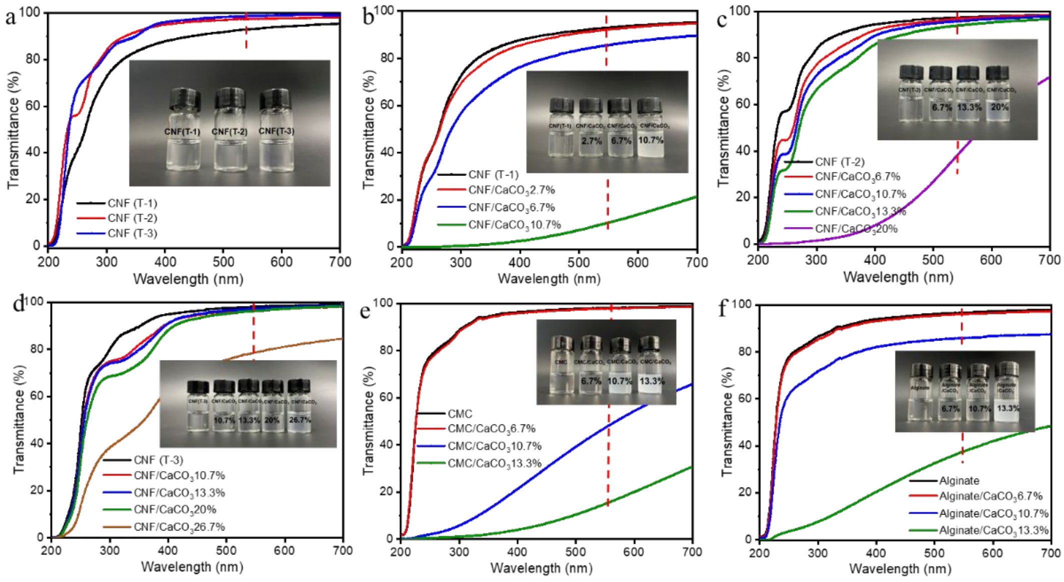

Figure S11. The light transmittance of (a) different oxidization times of as-prepared CNF suspensions (b) T-1/$CaCO_3$, (c) T-2/$CaCO_3$, (d)T-3/$CaCO_3$, (e) CMC/$CaCO_3$ and (f) alginate/$CaCO_3$ in the wavelength range of 200-700 nm.

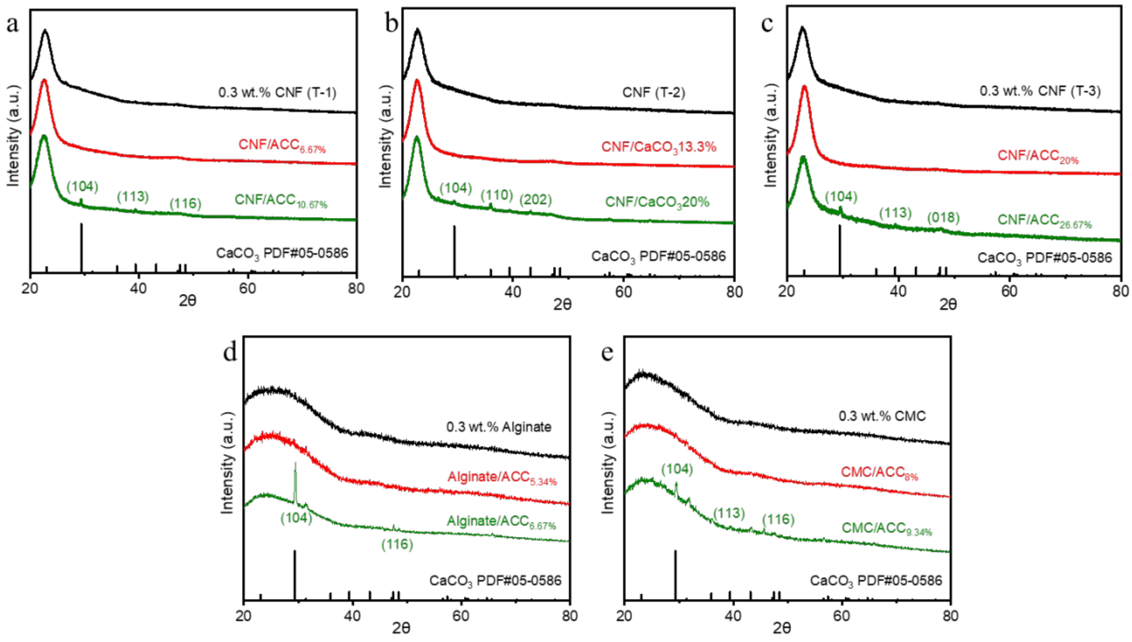

Figure S12. XRD patterns of as-prepared (a) T-1/$CaCO_3$, (b) T-2/$CaCO_3$, (c)T-3/$CaCO_3$, (d) alginate/$CaCO_3$ and (e) CMC/$CaCO_3$.

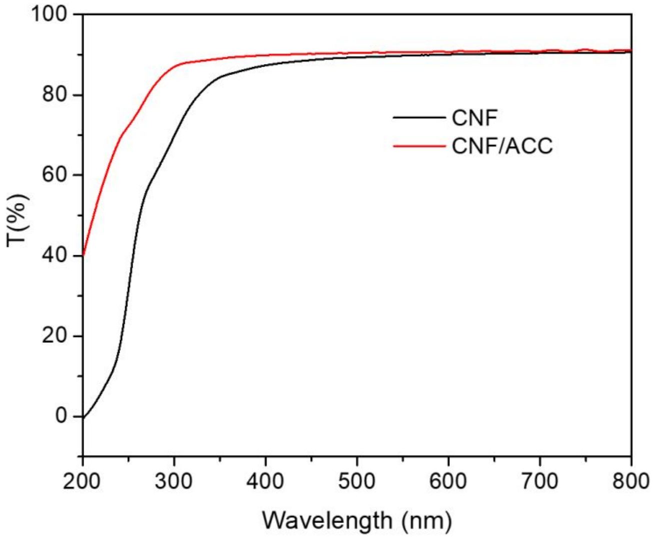

Figure S13. UV-vis spectra of CNF and CNF/ACC film.

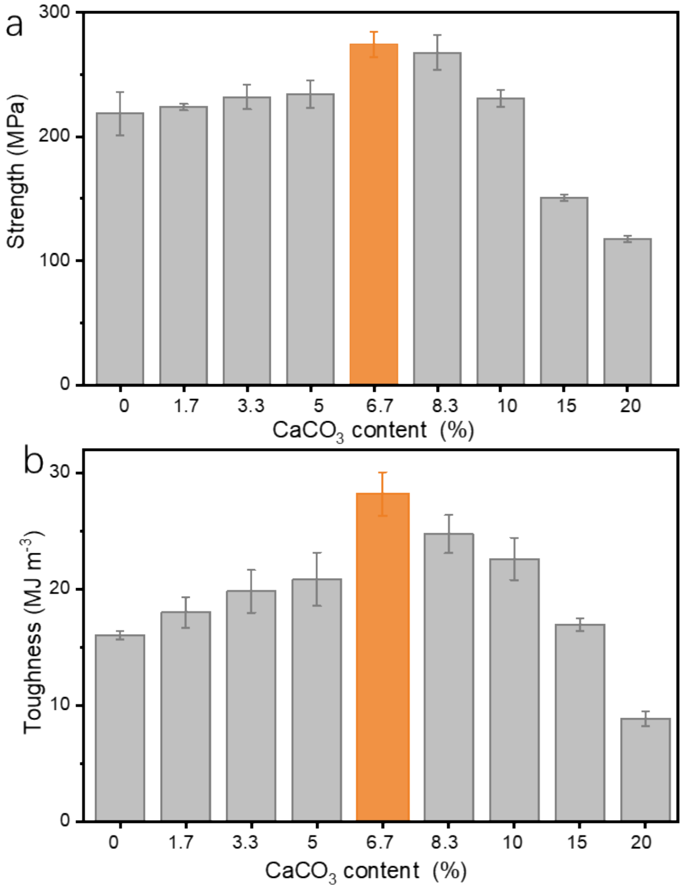

Figure S14. Strength and toughness of CNF/ACC composite films with different $CaCO_3$ content in wt.%.

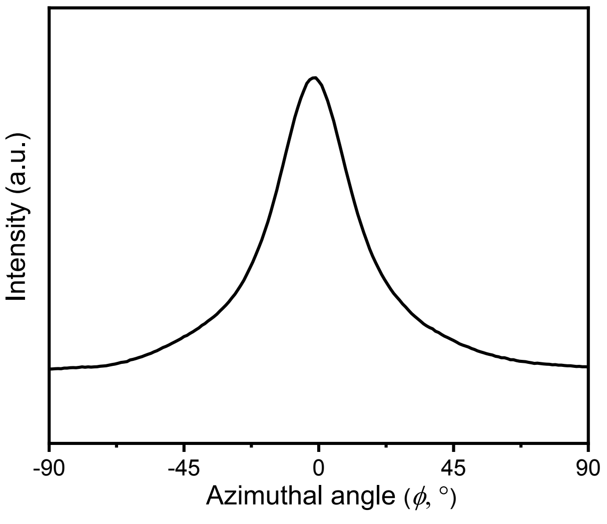

Figure S15. Azimuthal plot for the (101) peak of the 2D XRD Pattern of the CNF/ACC film in the range of -90° and 90°.

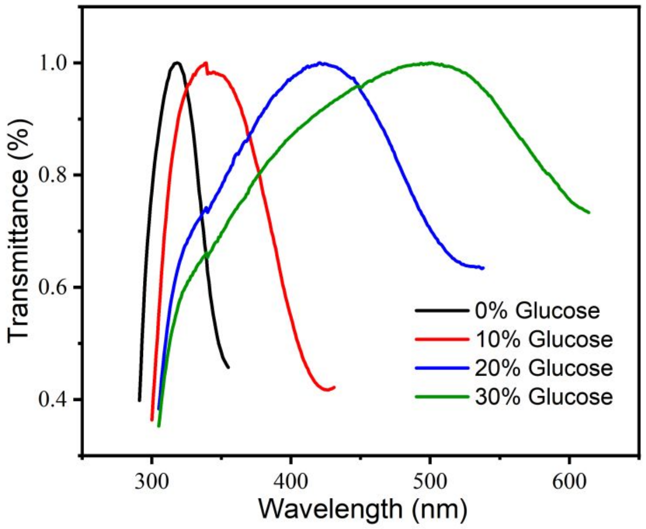

Figure S16. UV-vis spectra of sandwiched photonic film with different amount of glucose loading.

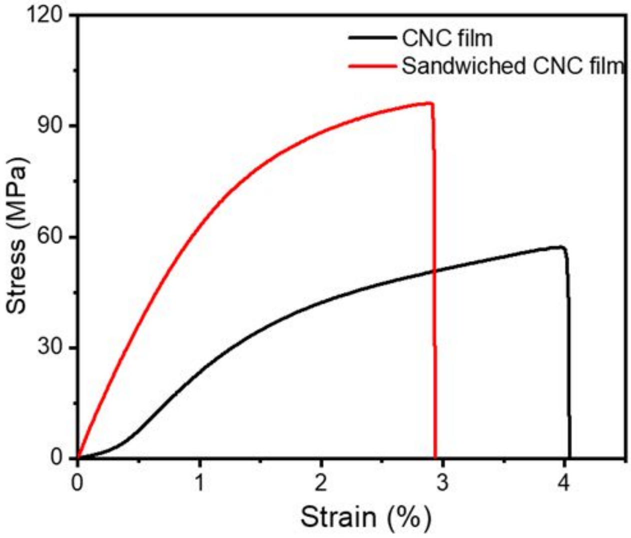

Figure S17. Stress-strain curves of CNC and sandwiched CNC film.

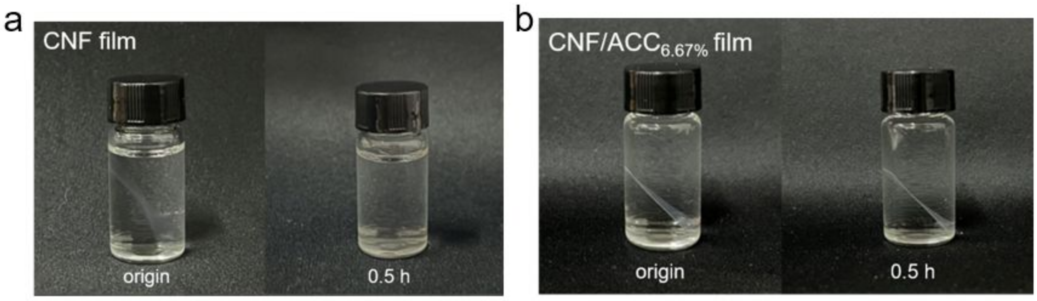

Figure S18. (a) The CNF and (b) CNF/ACC film in water for 0.5 h.

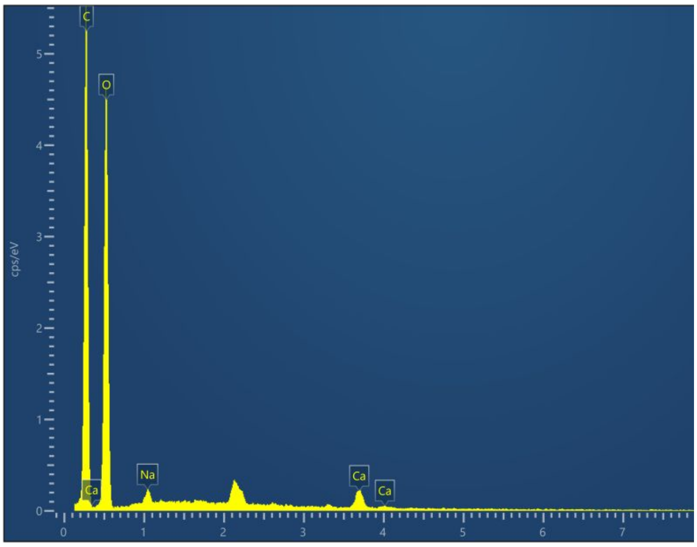

Figure S19. EDS spectra of CNF/$ACC_{6.67\%}$ film.

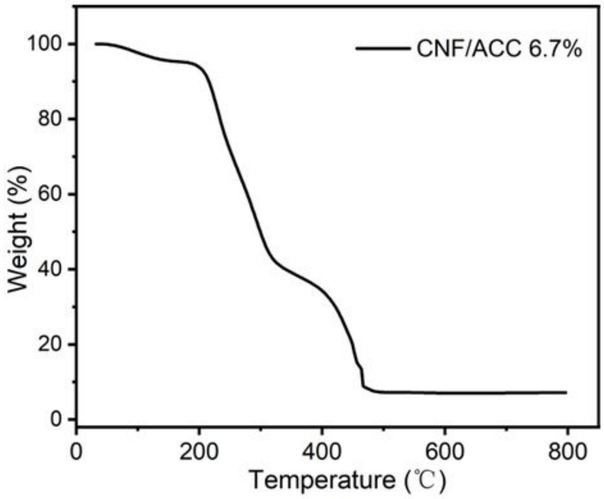

Figure S20. TGA curve of CNF/$ACC_{6.67\%}$ film.

Table S1. The mechanical properties of natural mineralized composites and reported biopolymer-calcium carbonate/phosphate composites.

	Strength (MPa)	Toughness (MJ/m^3)	Ref.
PVA/Alginate/HAP	120	21.5	[2]
PVA/Alginate/HAP	20	3	[3]
CNF/PAA/ACC	169	4	[4]
Gelatin/calcite	97	12	[5]
CNF/CaP	261	6.4	[6]
Alginate/brushite	267	8.7	[7]
Amyloid/HAP	12	0.1	[8]
Silk/HAP	100	1.5	[9]
Chitosan/Calcite	44	0.7	[10]
Lobster Exoskeleton	49	0.17	[11]
Crab Exoskeleton	31	1.02	[12]
Nacre	80	0.6	[13]
Compact Bone	117	1.2	[14]
Dentin	105	2.4	[15]
CNF/ACC	286	28.2	This work

References

(1) Saito, T.; Kimura, S.; Nishiyama, Y.; Isogai, A. Cellulose Nanofibers Prepared by TEMPO-Mediated Oxidation of Native Cellulose. *Biomacromolecules* **2007**, *8* (8), 2485–2491. https://doi.org/10.1021/bm0703970.

(2) Yu, Y.; Kong, K.; Tang, R.; Liu, Z. A Bioinspired Ultratough Composite Produced by Integration of Inorganic Ionic Oligomers within Polymer Networks. *ACS Nano* **2022**, *16* (5), 7926–7936. https://doi.org/10.1021/acsnano.2c00663.

(3) Yu, Y.; Guo, Z.; Zhao, Y.; Kong, K.; Pan, H.; Xu, X.; Tang, R.; Liu, Z. A Flexible and Degradable Hybrid Mineral as a Plastic Substitute. *Adv. Mater.* **2022**, *34* (9),

2107523. https://doi.org/10.1002/adma.202107523.
(4) Saito, T.; Oaki, Y.; Nishimura, T.; Isogai, A.; Kato, T. Bioinspired Stiff and Flexible Composites of Nanocellulose-Reinforced Amorphous CaCO3. *Mater. Horiz.* **2014**, *1* (3), 321–325. https://doi.org/10.1039/C3MH00134B.
(5) Li, X. Q.; Zeng, H. C. Calcium Carbonate Nanotablets: Bridging Artificial to Natural Nacre. *Adv. Mater.* **2012**, *24* (47), 6277–6282. https://doi.org/10.1002/adma.201202733.
(6) Yao, J.; Fang, W.; Guo, J.; Jiao, D.; Chen, S.; Ifuku, S.; Wang, H.; Walther, A. Highly Mineralized Biomimetic Polysaccharide Nanofiber Materials Using Enzymatic Mineralization. *Biomacromolecules* **2020**, *21* (6), 2176–2186. https://doi.org/10.1021/acs.biomac.0c00160.
(7) Gao, H.-L.; Chen, S.-M.; Mao, L.-B.; Song, Z.-Q.; Yao, H.-B.; Cölfen, H.; Luo, X.-S.; Zhang, F.; Pan, Z.; Meng, Y.-F.; Ni, Y.; Yu, S.-H. Mass Production of Bulk Artificial Nacre with Excellent Mechanical Properties. *Nat. Commun.* **2017**, *8* (1), 287. https://doi.org/10.1038/s41467-017-00392-z.
(8) Li, C.; Born, A.-K.; Schweizer, T.; Zenobi-Wong, M.; Cerruti, M.; Mezzenga, R. Amyloid-Hydroxyapatite Bone Biomimetic Composites. *Adv. Mater.* **2014**, *26* (20), 3207–3212. https://doi.org/10.1002/adma.201306198.
(9) Ling, S.; Qin, Z.; Huang, W.; Cao, S.; Kaplan, D. L.; Buehler, M. J. Design and Function of Biomimetic Multilayer Water Purification Membranes. *Sci. Adv.* **2017**, *3* (4), e1601939. https://doi.org/10.1126/sciadv.1601939.
(10) Raut, H. K.; Schwartzman, A. F.; Das, R.; Liu, F.; Wang, L.; Ross, C. A.; Fernandez, J. G. Tough and Strong: Cross-Lamella Design Imparts Multifunctionality to Biomimetic Nacre. *ACS Nano* **2020**, *14* (8), 9771–9779. https://doi.org/10.1021/acsnano.0c01511.
(11) Fabritius, H.-O.; Sachs, C.; Triguero, P. R.; Raabe, D. Influence of Structural Principles on the Mechanics of a Biological Fiber-Based Composite Material with Hierarchical Organization: The Exoskeleton of the Lobster Homarus Americanus. *Adv. Mater.* **2009**, *21* (4), 391–400. https://doi.org/10.1002/adma.200801219.
(12) Chen, P.-Y.; Lin, A. Y.-M.; McKittrick, J.; Meyers, M. A. Structure and Mechanical Properties of Crab Exoskeletons. *Acta Biomater.* **2008**, *4* (3), 587–596. https://doi.org/10.1016/j.actbio.2007.12.010.
(13) Barthelat, F.; Tang, H.; Zavattieri, P. D.; Li, C.-M.; Espinosa, H. D. On the Mechanics of Mother-of-Pearl: A Key Feature in the Material Hierarchical Structure. *J. Mech. Phys. Solids* **2007**, *55* (2), 306–337. https://doi.org/10.1016/j.jmps.2006.07.007.
(14) Lin, C.-Y.; Kang, J.-H. Mechanical Properties of Compact Bone Defined by the Stress-Strain Curve Measured Using Uniaxial Tensile Test: A Concise Review and Practical Guide. *Materials* **2021**, *14* (15), 4224. https://doi.org/10.3390/ma14154224.
(15) Sano, H.; Takatsu, T.; Ciucchi, B.; Russell, C. M.; Pashley, D. H. Tensile Properties of Resin-Infiltrated Demineralized Human Dentin. *J. Dent. Res.* **1995**, *74* (4), 1093–1102. https://doi.org/10.1177/00220345950740041001.
